# A Plea for the More General Use of Anæsthesia in Veterinary Surgery1Read before the thirty-sixth annual meeting of the American Veterinary Medical Association, New York City, September, 1899.

**Published:** 1899-11

**Authors:** J. P. Turner

**Affiliations:** Washington, D. C.


### A PLEA FOR THE MORE GENERAL USE OF ANÆSTHESIA IN VETERINARY SURGERY.[1]

By J. P. Turner, V.M.D.,
WASHINGTON, D. C.

In offering this paper I must crave your indulgence, as I have nothing new or original to offer, and to many of you my remarks apply to your daily practice. It is my purpose to ask you to seriously consider the humane side of surgery, although I feel most unworthy to bring up this subject, for there are with us surgeons of maturer years who have won their spurs and almost forgotten the winning thereof, while we of a younger generation are still wrestling with the ever-fascinating subject of surgery.

Chloroform was discovered by Samuel Guthrie, of Sackett's Harbor, New York, in 1831. Its effect upon animals was studied by Dr. Glover in 1842, and it was first used upon man by Dr. Simpson in 1847. Ether was first used by Dr. Morton in 1846, but both of these anæsthetics were first brought into general use during the Civil War.

In all the experiments made by the discoverers of these two great boons to humanity, the dog was the subject constantly used, and not until its use was perfected upon our own subjects was it tried upon man for surgical purposes; and yet forty years after these studies were perfected do we see some veterinary surgeons still pursuing the methods of the ancients, and by neatly devised straps and appliances perpetrate the most excruciating pain on a helpless subject.

I use the word "some" cautiously, yet we know that this word covers more of the rank and file of the profession than it should.

While we are proud to observe that our best operators are now using anæsthesia in most of their major operations, yet those of you who have had the benefit of extensive travel and experience among the profession at large know that the chloroform-bag is seldom seen and more rarely used.

The result has been, and the feeling still exists, largely among the laity, that we are a hard-hearted profession, and undoubtedly we are lowered to a certain extent in their estimation; and how can their feelings be otherwise when they are so frequently entertained by the sight of serious and painful operations, often extend-

[1] Read before the thirty-sixth annual meeting of the American Veterinary Medical Association, New York City, September, 1899.

ing over a long period, with a struggling, groaning animal neatly tied up with hopple and side-line.

How many of us have sat by the hour at the feet of some learned professor and seen wonderfully skilled operations performed, and listened to his words of wisdom in surgical clinics; yet many of us could never consider such work worthy of much commendation while the poor brute groaned and fought from the acute pain of such operations as the major punctured sole operation, or the long, deep incisions in fistulous withers, or the frightful pain of neurectomy, when the near-sighted operator would pinch the digital nerve with his forceps, to distinguish it from the too neighborly artery; and all this cruelty with plenty of chloroform, ether, and cocaine at hand.

Especially distasteful was this practice to those of us who were in the habit of learning surgical methods at the clinics of the medical hospitals, and we could hardly help notice and comment upon the different methods of the medical and veterinary surgeon when we observed the clean incisions, rapidly ligated arteries on the anæsthetized man, and the struggling horse or dog, with an operator more or less excited, cutting ragged incisions between kicks, and the blood spurting over everybody within reach, owing to the difficulty of ligating arteries on such a lively subject.

Why should some veterinary surgeons persist in stringing bitches to ladders and spaying them roughly, cruelly, and in a most unscientific manner, when five cents' worth of chloroform, carefully administered, or ten cents' worth of ether, not so carefully given, would relieve the brute of intense pain and suffering and permit the performance of a better operation?

Or what reason have we to castrate without the use of anæsthetics save that it is the accustomed way, or the method we were taught? The highest ambition of any surgeon should be to relieve pain and not cause it. Who is to blame for this existing condition? I say without reserve the veterinary colleges, for they have not taught this branch of surgery as they should in the past. A few years ago men graduated from our best colleges who had never seen a horse or cow anæsthetized. Occasionally a dog was given ether, but the hippo-lasso or hopple was reserved for the horse; the result was that young and inexperienced surgeons dreaded to anæsthetize, fearful of sudden, fatal results. At least that was my feeling ten years ago, when I first placed my crude, endless nose-gag into a horse's mouth and began the administration of chloroform, fully expecting a fatal termination and a ruined reputation; but with success and experience this feeling passes away. I note with great pleasure that many of the colleges are now teaching students the use of anæsthetics, as their use is absolutely necessary in the successful performance of some of our modern operations.

Now consider some of the so-called objections to the use of anæsthesia, particularly of horses and cows. The expense of an old nose-bag and from two to eight ounces of chloroform is too trivial to be any valid excuse. The most frequent objection to their use by those who do not use anæsthetics is that their successful use requires an expert assistant. This is not a tenable excuse. Use an expert when you can, otherwise go it alone. When properly given by a cool operator there will be sufficient time for any ordinary operation after you have anæsthetized your patient, and all that is necessary is the occasional observance of the respirations. I have had ample experience to defend this assertion. We are especially favored in veterinary surgery, owing to the well-known effect of chloroform upon animals, since death occurs almost invariably from arrest of respiration, due to the abolition of the function-power of the medulla oblongata, and so seldom is death due to cardiac arrest when the chloroform is given in excess that many authorities have denied it altogether. The practical point to be learned is that the operator can, by occasionally noting the respirations, determine exactly the patient's condition. With ether, the supposed danger of cardiac arrest is eliminated, owing to the stimulant effect of ether, but with ether the appearance of very slow, or interrupted respirations, indicates that we are carrying the anæsthesia too far.

I claim the advantages gained by the use of anæsthetics far outweigh the supposed danger of their use. The struggling which always occurs at first is only momentary, seldom lasting more than two or three minutes, so that the danger of a fractured vertebra in the horse only lasts during the preliminary period of excitement, while in the ordinary operation without anæsthesia it is always present. Furthermore, the operation can be performed more rapidly and better on the quieted subject, and the danger to the operator is the minimum; and this should always be considered as a paramount duty by every veterinary surgeon, as our journals are continually noting serious accidents, and often deaths, through lack of proper precaution and the absence of either general or local anæsthesia. Unfortunately, we have but few statistics in veterinary literature on any subject. In five hundred operations upon dogs of all ages and breeds, in which chloroform was used as the anæsthetic, Dr. Hobday, of London, lost but one patient, an old, fat pug, which showed post-mortem a rupture of the portal vein. Operations upon cats are more dangerous where chloroform is used, as the same authority lost three out of one hundred and twenty operations. Hobday ascribes his success with chloroform to the use of a certain form of inhaler, described by him in current veterinary literature, this inhaler allowing a plentiful admixture of air with the chloroform—this being the secret of its successful use on all animals, and in many of our accidents with chloroform we are the erring party and not the drug. With ether all is different, and we must rigidly exclude the air. According to Dr. H. C. Wood, chloroform is fatal in 1 out of 5860 cases; ether in 1 out of 16,542 cases where administered to the human.

I never use chloroform on dogs, as I believe ether is much safer. Even with it death occurs occasionally from œdema of the glottis. I have lost two patients in this manner, one dying very quickly, in spite of artificial respiration and hypodermatic stimulants; the other was revived, but died the following day, the death being due to some alcohol given the dog as he was reviving, by an assistant, the alcohol entering the lungs instead of the stomach, causing death from œdema of the entire bronchial tract. Both of these were pups less than six months old; in older dogs there is but little danger. Each surgeon has his particular method of using anæsthetics, and thinks his is the only way, because he is more expert in its use. I still cling to the nose-bag, described by Fleming, made of stiff leather, with canvas ends, both being open and each having a drawing-string.

This bag is placed in the mouth, the upper string drawn together and tied to the nose-band of the halter, it being understood that the horse is prone and properly secured, and a blanket folded around the upper part of the head, both for protection and to exclude the light from the eyes. The tongue is drawn to one side and one-half ounce of chloroform is sprinkled upon a handkerchief and inserted into the bag, the distal end of which is kept widely open. Usually the animal struggles, when the chloroform should be withdrawn, three or four inspirations allowed, then the chloroform is replaced and the anæsthesia quietly and slowly accomplished. The chloroform is renewed by one-half ounce doses as needed, usually in from one and a half to two minutes, depending upon the behavior of the animal. Usually two to four ounces suffice, but as much as eight ounces have been required to bring them into the operative stage, which is indicated by failure to retract the tongue, partial relaxation and failure to notice the prick of a pin. I am not in favor of large doses to get rapid anæsthesia, as it is a dangerous procedure. The animal needs plenty of air with the chloroform, at least one-half.

As the operative stage approaches two or three ounces of ether should be poured upon the handkerchief and the bag almost closed, and the operator can proceed. If the effects of the anæsthetic wear away, as indicated by movements of the animal, the man at the head can pour in more ether, the only precaution required is for the operator to occasionally watch the respirations. By this method of bringing on anæsthesia with chloroform and continuing it with ether, a horse or cow can be kept under its influence for an hour very readily. The two drugs should never be mixed and thus given together, owing to their different degrees of volatility. All instruments and dressings should be prepared previously to casting. A good surgeon is always prepared for emergencies, hence the advisability of having stimulants handy, preferably aqua ammonia, for intravenous use and inhalation, or both if necessary. Cocaine intravenously is a splendid respiratory stimulant.

The introduction of cocaine as a practical local anæsthetic by Dr. Karl Koller, in 1884, has proved a boon to the progressive veterinary surgeon. By its use we are now able to render painless such operations as firing, tenotomy, myotomy, neurectomy, and the removal of superficial tumors. With the use of cocaine or eucaine it is seldom necessary in these operations to cast an animal, excepting those which are very nervous. The hypodermatic injection of either drug (3 or 4 per cent. solution in sodium chloride, same strength) over the point of operation produces anæsthesia very rapidly. Flexor tendons can be fired painlessly by injecting this solution on both the internal and external plantar nerves, just below the knee. Likewise splints, spavins, and curbs are fired painlessly. The use of cocaine in neurectomy is general. I ascribe the occasional bad healing of wounds where cocaine has been used to an infected syringe as much, if not more, than to the supposed congestion following the use of cocaine. Its use is even as successful, when freely used, around a fistulous wither, or poll-evil. By studying close and frequently using these local anæsthetics we can avoid much of the hard labor, anxiety, and danger of throwing horses for operations.

In closing, I thank you for your patience in listening to these old, culled-over, unoriginal facts, and I am amply repaid if I may cause some discussion on this branch of surgery which will enlighten us more and will lead to the more general adoption of humane principles in surgery through the more frequent use of anæsthetics.

